# Quality of End-of-Life Care for Vietnam Veterans: Implications for Practice and Policy

**DOI:** 10.1093/geroni/igaa057.793

**Published:** 2020-12-16

**Authors:** Joan Carpenter, Dawn Smith, Hilary Griffin, Daniel Kinder, Joshua Thorpe, Mary Ersek, Ann Kutney-Lee

**Affiliations:** 1 Department of Veterans Affairs, Berlin, Maryland, United States; 2 Department of Veterans Affairs, Philadelphia, Pennsylvania, United States

## Abstract

In federal response to the aging population of Vietnam-era Veterans, Congress directed the Department of Veterans Affairs (VA) to create a pilot program to identify and develop best practices for improving hospice care for this population. A first step in VA’s response was to identify whether the end-of-life (EOL) care needs and outcomes of Vietnam-era Veterans differed from previous generations. Using medical records and bereaved family surveys, we examined clinical characteristics, healthcare utilization, and EOL quality indicators for Vietnam-era Veterans who died in VA inpatient settings between 2013-17. Contemporaneous comparisons were made with World War II/Korean War-era Veterans. Compared to prior generations, higher percentages of Vietnam-era Veterans had mental health/substance use diagnoses and disability. Similar percentages of family members in both groups reported that overall EOL care was excellent; however, post-traumatic stress disorder management ratings by families of Vietnam-era Veterans were significantly lower. Although current VA EOL practices are largely meeting the needs of Vietnam-era Veterans, greater focus on mental health comorbidity, including post-traumatic stress disorder, Agent Orange-related conditions, and ensuring access to quality EOL care in the community is warranted. Policymakers and healthcare professionals should anticipate more physical and mental health comorbidities among Veterans at EOL as Vietnam-era Veterans continue to age. Findings are being used to inform the development of standardized EOL care protocols and training programs for non-VA healthcare providers that are tailored to the needs of this population.

